# Estimating malaria parasite prevalence from community surveys in Uganda: a comparison of microscopy, rapid diagnostic tests and polymerase chain reaction

**DOI:** 10.1186/s12936-015-1056-x

**Published:** 2015-12-30

**Authors:** Joaniter I. Nankabirwa, Adoke Yeka, Emmanuel Arinaitwe, Ruth Kigozi, Chris Drakeley, Moses R. Kamya, Bryan Greenhouse, Philip J. Rosenthal, Grant Dorsey, Sarah G. Staedke

**Affiliations:** Department of Medicine, Makerere University College of Health Sciences, Kampala, Uganda; Infectious Diseases Research Collaboration, Kampala, Uganda; Makerere University School of Public Health, College of Health Sciences, Kampala, Uganda; London School of Hygiene and Tropical Medicine, London, UK; School of Medicine, Makerere University College of Health Sciences, Kampala, Uganda; Department of Medicine, University of California San Francisco, San Francisco, USA

**Keywords:** Malaria, Parasite prevalence, Community surveys, Surveillance, Diagnostics, Rapid diagnostic tests, Microscopy

## Abstract

**Background:**

Household surveys are important tools for monitoring the malaria disease burden and measuring impact of malaria control interventions with parasite prevalence as the primary metric. However, estimates of parasite prevalence are dependent on a number of factors including the method used to detect parasites, age of the population sampled, and level of immunity. To better understand the influence of diagnostics, age, and endemicity on estimates of parasite prevalence and how these change over time, community-based surveys were performed for two consecutive years in three settings and the sensitivities of microscopy and immunochromatographic rapid diagnostic tests (RDTs) were assessed, considering polymerase chain reaction (PCR) as the gold standard.

**Methods:**

Surveys were conducted over the same two-month period in 2012 and 2013 in each of three sub-counties in Uganda: Nagongera in Tororo District (January–February), Walukuba in Jinja District (March–April), and Kihihi in Kanungu District (May–June). In each sub-county, 200 households were randomly enrolled and a household questionnaire capturing information on demographics, use of malaria prevention methods, and proxy indicators of wealth was administered to the head of the household. Finger-prick blood samples were obtained for RDTs, measurement of hemoglobin, thick and thin blood smears, and to store samples on filter paper.

**Results:**

A total of 1200 households were surveyed and 4433 participants were included in the analysis. Compared to PCR, the sensitivity of microscopy was low (65.3 % in Nagongera, 49.6 % in Walukuba and 40.9 % in Kihihi) and decreased with increasing age. The specificity of microscopy was over 98 % at all sites and did not vary with age or year. Relative differences in parasite prevalence across different age groups, study sites, and years were similar for microscopy and PCR. The sensitivity of RDTs was similar across the three sites (range 77.2–82.8 %), was consistently higher than microscopy (p < 0.001 for all pairwise comparisons), and decreased with increasing age. The specificity of RDTs was lower than microscopy (76.3 % in Nagongera, 86.3 % in Walukuba, and 83.5 % in Kihihi) and varied significantly by year and age. Relative differences in parasite prevalence across age groups and study years differed for RDTs compared to microscopy and PCR.

**Conclusion:**

Malaria prevalence estimates varied with diagnostic test, age, and transmission intensity. It is important to consider the effects of these parameters when designing and interpreting community-based surveys.

## Background

In the last decade, widespread scale-up of malaria interventions including long-lasting insecticide-treated bed nets (ITNs), indoor residual spraying of insecticides, intermittent preventive treatment in pregnancy, and prompt and effective treatment with artemisinin-based combination therapy, has substantially reduced the malaria burden in Africa and elsewhere [1, 2]. However, coverage of malaria control interventions varies, and the burden of malaria remains high in some countries, including Uganda [3–5]. Malaria surveillance, monitoring, and evaluation are critical for estimating disease burden.

Population-based household surveys are one of the primary tools for monitoring malaria disease burden and measuring impact of malaria control interventions. Such surveys include the Demographic and Health Survey (DHS) [6], the Multiple Indicator Cluster Survey (MICS) [7], and the Malaria Indicator Survey (MIS) [8]. The primary metric for estimating malaria burden from these surveys is parasite prevalence, which is a simple measurement of the proportion of individuals in a representative sample who have malaria parasites detectable in their blood at a given point in time [9, 10]. Despite the widespread use of this metric, estimates of parasite prevalence are dependent on a number of factors, including the method used to detect parasites, the age of the population sampled, and the underlying immunity of the population (which is dependent on both endemicity and age, as a proxy for exposure) [11–13]. Several types of malaria diagnostic tests are available, including microscopic evaluation of Giemsa-stained blood smears, immunochromatographic rapid diagnostic tests (RDTs) and polymerase chain reaction (PCR) assays, which are all fundamentally different tests. Historically, microscopy has been used most commonly to diagnose malaria, but limited sensitivity for detecting low-level parasitaemia and the need for skilled microscopists are disadvantages of this method. RDTs require less technical skill and have become widely available, but may lack specificity; RDTs that identify histidine rich protein II (HRP-2), a parasite antigen that may circulate for weeks following successful malaria treatment, may be falsely positive due to recent prior infection [14]. Molecular amplification techniques, such as PCR, offer improved sensitivity, but are not widely used outside of research settings due to high cost and technical requirements. Estimates of parasite prevalence also have a complex relationship with age and endemicity, and test results may be influenced by host immunity and recent anti-malarial treatment [14–16].

To better understand the influence of diagnostics, age, and endemicity on estimates of parasite prevalence and how these change over time, community-based surveys were performed for two consecutive years in three settings and the sensitivities of microscopy and rapid diagnostic tests (RDTs) were assessed, considering polymerase chain reaction (PCR) as the gold standard.

## Methods

### Study setting and time of the surveys

Surveys were conducted over the same two-month period in 2012 and 2013 in each of three sub-counties; Nagongera in Tororo District (January–February), Walukuba in Jinja District (March–April), and Kihihi in Kanungu District (May–June). These sub-counties were selected to represent varied malaria transmission intensity in Uganda: annual entomological inoculation rates were estimated in 2011–2013 to be 310, 2.8, and 32 infective bites per person year in Nagongera, Walukuba, and Kihihi, respectively [17]. No major malaria control interventions were implemented between 2012 and 2013 in any of the sub-counties.

### Study design, population and procedures

Details of these cross-sectional surveys have been described previously [15]. Briefly, in 2011 all households at the three sites were enumerated and mapped to generate a sampling frame. For each survey, households were randomly selected from the enumeration list and sequentially screened until 200 households were enrolled. The purpose of the study was discussed with the head of the household or their designate and consent to participate in the survey was sought. Households with no adult respondent during the initial contact were re-visited up to three times before excluding them from the sample selection. Households were also excluded if the house was vacant or the head of the household refused to provide informed consent.

Following consent, a household questionnaire was administered to the head of the household or their designate. The questionnaire was used to capture information on demographics of all household members, use of malaria prevention methods, and proxy indicators of wealth. Finger prick blood samples were obtained from all children under 15 years and one randomly selected household member from five age categories (15–24, 25–34, 35–44, 45–54, and ≥55 years) for RDT testing, thick and thin blood smears, and to store on filter paper.

### Laboratory evaluations

RDT testing was performed in the field by the trained laboratory technicians, using SD Bioline Malaria Ag P.f., which detects histidine-rich protein II (HRP-II) of *Plasmodium falciparum*. The RDTs were obtained from Standard Diagnostics, Inc, were used before the expiration date, and were transported and stored according to the recommended storage conditions (temperature 4–30° C, avoid humidity). Tests were kept in their original packaging at room temperature and were prepared using approximately 5 µl of blood and read according to the manufacturer’s instructions. Participants who were RDT positive were treated with artemether-lumefantrine.

Thick and thin smears were prepared using between 5 µl and 10 µl of blood. They were stained with 2 % Giemsa for 30 min and read by expert microscopists who were blinded to the RDT results. Thick smears were evaluated for presence of parasites and gametocytes. Parasite densities were determined by counting the number of parasites per 200 leukocytes (or per 500, if the count was less than 10 parasites per 200 leukocytes), assuming a leukocyte count of 8000 cells/µl. Asexual parasitaemia of any level was reported as positive and a smear was considered negative after reviewing 100 high powered fields. Gametocytaemia was determined using similar methodology. All positive thick smears had their corresponding thin smears viewed for species identification. Two independent and experienced microscopists read all slides, with a third microscopist resolving any discrepancies. The expertise level of the microscopists according to the WHO competency assessment protocol is estimated to be level III.

Blood was spotted onto filter paper (Whatman 3MM: Whatman, Maidstone, UK), allowed to dry overnight and stored at −20 °C with desiccant for PCR testing. If a sample was negative by microscopy and/or RDT, polymerase chain reaction was performed to detect the presence of *P. falciparum*. DNA was extracted from filter paper samples by use of chelex resin and parasites detected using nested PCR targeting the 18S rRNA gene as previously described [18]. Sample collection and analysis for all diagnostic tools was performed by trained laboratory technicians and was standardized across the different surveys with the guidance of standard operating procedures. The same laboratory procedures were used for samples from all three sites for both annual surveys.

### Data management and statistical analysis

Data were collected using hand-held computers which were programmed to include range checks, structure checks and internal consistency checks. Statistical analysis was performed using Stata version 12 (STATA Corporation, College Station, TX, USA). Measures of diagnostic accuracy (sensitivity, specificity, positive predictive value and negative predictive values) were calculated using PCR as the gold standard. For samples that were positive by microscopy and RDT, PCR testing was not performed and assumed to be positive. Sensitivity was defined as the proportion of test results that were positive by both PCR and the test of interest (either RDT or microscopy) divided by the total number of test results that were positive by PCR. Specificity was defined as the proportion of test results that were negative by both PCR and the test of interest, divided by the total number that were negative by PCR. Positive predictive value was defined as the proportion of test results that were positive by both PCR and the test of interest, divided by the total number of tests that were positive by the test of interest. Negative predictive value was defined as the proportion of test results that were negative by both PCR and test of interest, divided by the total number of tests that were negative by the test of interest. Comparison of estimates of parasite prevalence by microscopy and RDT with PCR stratified by age groups and year were made using McNemar’s *X*^2^ test. Associations between age groups and year with estimates of parasite prevalence using different diagnostic modalities were made using multivariate log-binomial regression. Graphical presentation of the relationships between age and both sensitivity and specificity were made using Lowess smoothing with an upper limit of 40 years due to sparsity of data above this age cut-off. A *p* value <0.05 was considered statistically significant.

### Ethical approval and informed consent

Ethical approval was obtained from the Makerere University School of Medicine Research and Ethics Committee, the Uganda National Council of Science and Technology, the London School of Hygiene and Tropical Medicine Ethics Committee, and the University of California, San Francisco Committee on Human Research. Written consent to participate was sought from all participants.

## Results

### Study participants

**Fig. 1:**
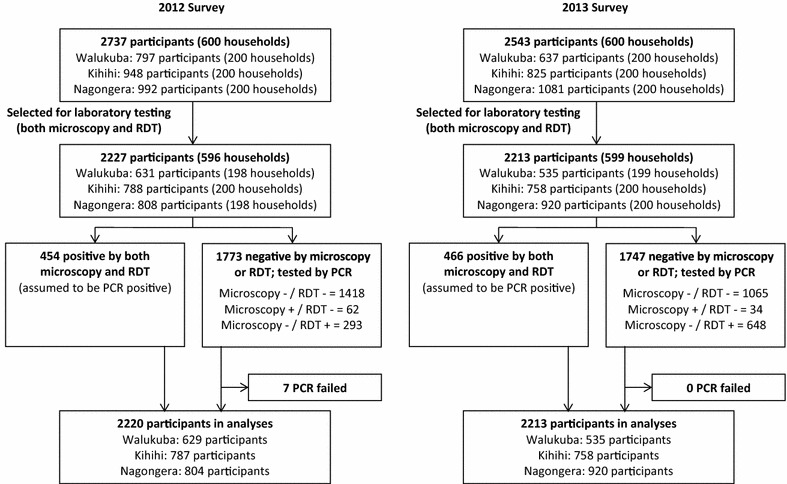
Study profile

A total of 1200 households were surveyed at the three sites, including 5280 participants. Of the enrolled participants, 4440 (84 %) were selected for laboratory testing, and PCR was performed on 3520, with the remaining 920 samples that were positive by both microscopy and RDT assumed to be PCR positive (Fig. 1).

Characteristics of study participants are presented in Table 1. Age and gender were similar across the three sites in both surveys. In 2012, ITN coverage was highest in Nagongera (52.6 %), followed by Walukuba (40.1 %) and Kihihi (31.5 %), with small decreases at all three sites in 2013. Based on thin smear readings, the prevalence of *P. falciparum* mono-infection was 93.1 %, mixed infection including *P. falciparum* 5.0 %, and non-falciparum infection 1.9 %, with no *Plasmodium vivax* infections detected. The prevalence of gametocytes detected by microscopy was higher in Nagongera than in Walukuba and Kihihi (p < 0.001 for both comparisons), with no significant change from 2012 to 2013 at any of the sites.

**Table 1:** Characteristics of study participants by study site and year

Characteristics	Walukuba		Kihihi		Nagongera	
	2012	2013	2012	2013	2012	2013
Number of participants	629	535	787	758	804	920
Female gender, n (%)	348 (55.3 %)	302 (56.5 %)	442 (56.2 %)	422 (55.7 %)	471 (58.6 %)	508 (55.2 %)
Median age in years (IQR^a^)	12 (5–25)	14 (5–25)	12 (5–28)	13 (5–26)	11 (6–28)	11 (5–25)
Age categories, n (%)						
<5 years	154 (24.5 %)	130 (24.3 %)	183 (23.3 %)	162 (21.4 %)	158 (19.7 %)	195 (21.2 %)
5–15 years	214 (31.0 %)	150 (28.0 %)	281 (35.7 %)	277 (36.5 %)	343 (42.7 %)	409 (44.5 %)
>15 years	261 (41.5 %)	255 (47.7 %)	323 (41.0 %)	319 (42.1 %)	303 (37.7 %)	316 (34.4 %)
ITN use^b^, n (%)	252 (40.1 %)	196 (36.6 %)	248 (31.5 %)	178 (23.5 %)	423 (52.6 %)	418 (45.4 %)
Geometric mean parasite density/µL^c^	430	656	827	2187	908	1880
Parasite ranges	16–32,800	48–19,200	16–74,240	16–98,680	16–139,480	16–188,080
Parasite species by thin smear^c^						
*P. falciparum*	91.7 %	92.3 %	92.4 %	82.1 %	95.7 %	93.2 %
*P. falciparum* + *P. malariae*	2.8 %	7.7 %	3.3 %	10.4 %	1.4 %	6.6 %
*P. falciparum* + *P. ovale*	0	0	0	0	1.4 %	0
*P. malariae*	5.6 %	0	4.3 %	7.5 %	1.1 %	0.3 %
*P. ovale*	0	0	0	0	0.3 %	0
Gametocytes present, n (%)	15 (2.4 %)	13 (2.4 %)	13 (1.7 %)	16 (2.1 %)	107 (13.3 %)	95 (10.3 %)

^a^Intra-quartile range

^b^Reported sleeping under an ITN the evening prior to the survey

^c^If positive by microscopy

### Diagnostic accuracy of microscopy and RDTs

**Fig. 2:**
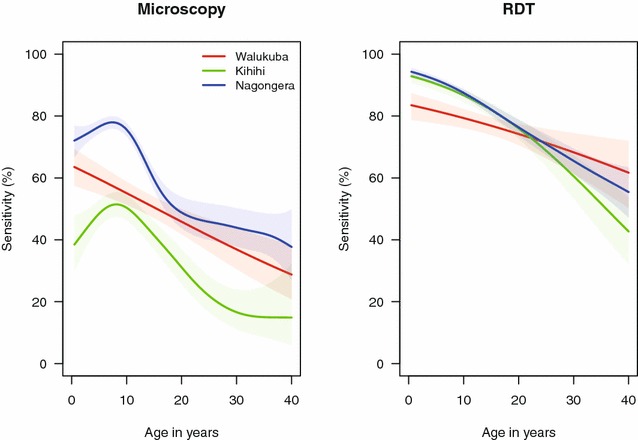
Sensitivity of microscopy and RDTs by age-groups at the three study sites

The sensitivity of microscopy was higher in Nagongera (65.3 %) compared to Walukuba (49.6 %, p < 0.001) and Kihihi (40.9 %, p < 0.001), and decreased from 2012 to 2013 in Nagongera (p < 0.001, Table 2). Importantly, sensitivity of microscopy fluctuated by a factor of 2 with age. In Nagongera and Kihihi, sensitivity increased until approximately 9 years of age, then decreased with increasing age; in Walukuba a linear decrease with age was observed, with granularity of this relationship possibly limited by the lower number of positive samples at this site (Fig. 2). The specificity of microscopy was over 98 % at all three sites in both years of the study and did not change appreciably with age (Table 2; Fig. 2).

**Table 2:** Diagnostic accuracy of microscopy and RDTs using PCR as the gold standard

Study site	Year	Number tested	Number positive	Measures of diagnostics accuracy (95 % CI)			
				Sensitivity	Specificity	PPV	NPV
Microscopy							
Walukuba	2012	629	72	50.4 % (41.5–59.3 %)	98.6 % (97.1–99.4 %)	90.3 % (81.0–96.0 %)	88.5 % (85.6–91.0 %)
	2013	535	52	48.5 % (38.6–58.6 %)	99.5 % (98.3–99.9 %)	96.2 % (86.8–99.5 %)	89.0 % (85.9–91.7 %)
Kihihi	2012	787	92	45.8 % (38.4–53.4 %)	98.4 % (97.0–99.2 %)	89.1 % (80.9–94.7 %)	86.0 % (83.2–88.5 %)
	2013	758	67	35.8 % (28.7–43.4 %)	99.3 % (98.2–99.8 %)	94.0 % (85.4–98.3 %)	83.6 % (80.7–86.3 %)
Nagongera	2012	804	351	71.8 % (67.5–75.7 %)	99.1 % (97.3–99.8 %)	99.1 % (97.5–99.8 %)	69.8 % (65.3–74.0 %)
	2013	920	381	60.3 % (56.3–64.1 %)	98.3 % (96.1–99.4 %)	98.7 % (97.0–99.6 %)	54.0 % (49.7–58.3 %)
RDT							
Walukuba	2012	629	124	77.5 % (69.3–84.4 %)	95.2 % (92.9–96.9 %)	80.6 % (72.6–87.2 %)	94.3 % (91.9–96.1 %)
	2013	535	183	76.7 % (67.3–84.5 %)	75.9 % (71.6–79.9 %)	43.2 % (35.9–50.7 %)	93.2 % (90.0–95.6 %)
Kihihi	2012	787	178	78.8 % (72.0–84.5 %)	93.9 % (91.7–95.7 %)	79.2 % (72.5–84.9 %)	93.8 % (91.5–95.5 %)
	2013	758	311	85.8 % (79.7–90.6 %)	72.5 % (68.7–76.1 %)	48.6 % (42.9–54.3 %)	94.4 % (91.9–96.3 %)
Nagongera	2012	804	444	79.4 % (75.5–82.9 %)	81.5 % (76.8–85.6 %)	86.7 % (83.2-89.7 %)	72.2 % (67.3–76.8 %)
	2013	920	620	85.4 % (82.4– 88.1 %)	70.6 % (65.1–75.7 %)	86.0 % (83.0–88.6 %)	69.7 % (64.1–74.8 %)

Sensitivity is the percentage of test results that are positive by both PCR and test of interest (RDT or Microscopy) divided total positive by PCR. Specificity is the percentage of test results that are negative by both PCR and test of interest (RDT or Microscopy) divided total negative by PCR

**Fig. 3:**
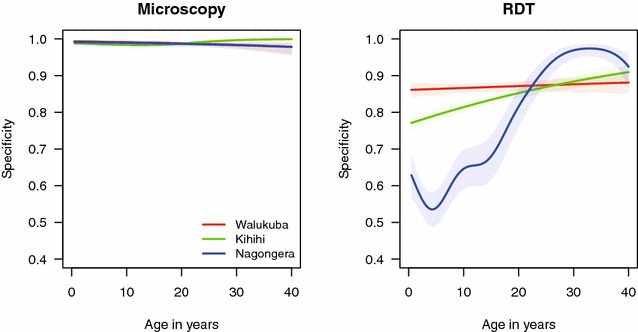
Specificity of microscopy and RDTs by age-groups at the three study sites

The sensitivity of RDTs was similar across the three sites (range 77.2–82.8 %) and consistently higher than microscopy (p < 0.001 for all pairwise comparisons). The sensitivity of RDTs did not change significantly from 2012 to 2013 in Walukuba and Kihihi, but increased significantly in Nagongera (p = 0.008, Table 2). Similar to microscopy, the sensitivity of RDTs decreased with increasing age (Fig. 2). The specificity of RDTs was lower than microscopy at all three sites and, in contrast to microscopy, varied by site, year, and age. Specificity was significantly lower in Nagongera compared to Walukuba and Kihihi (p < 0.001 for both), as might be expected given the very high transmission intensity in Nagongera. Unexpectedly, the specificity of RDTs decreased dramatically at all three sites from 2012 to 2013 (p < 0.001 in Walukuba and Kihihi, p = 0.002 in Nagongera, Table 2). The relationship between the specificity of RDTs and age differed across the three sites; in Walukuba, specificity did not change appreciably with age, while in Kihihi and Nagongera specificity increased with increasing age, with the degree of change much greater in Nagongera (Fig. 3).

### Comparisons of estimates of parasite prevalence by microscopy and RDTs

As expected, estimates of parasite prevalence by microscopy were consistently lower than estimates by PCR in all sites, all age-groups and in both years of the survey (Table 3). In contrast, estimates of parasite prevalence by RDT showed a complex pattern of variation in comparison to PCR when stratified by site, age and year (Table 3). In Walukuba, parasite prevalence by RDT was significantly higher than by PCR in the youngest and oldest age groups, but similar in the middle age group. In Kihihi, parasite prevalence by RDT was significantly higher than by PCR in all age groups. In Nagongera, parasite prevalence by RDT was significantly higher than by PCR in the youngest age group, similar in the middle age group and significantly lower in the oldest age group. At all three sites, parasite prevalence by RDT in relation to PCR increased from 2012 to 2013, resulting in the decrease in specificity noted above.

**Table 3:** Comparison of estimates of parasite prevalence by microscopy and RDTs with PCR stratified by age and year

Study site	Covariate		N	PCR	Microscopy			RDT		
				PP^a^	PP^a^	PR^b^ (95 % CI)	p-value	PP^a^	PR^b^ (95 % CI)	p-value
Walukuba	Age categories	<5 years	284	9.9 %	6.3 %	0.64 (0.46–0.89)	0.01	22.9 %	2.32 (1.71–3.16)	<0.001
		5–15 years	364	30.5 %	18.1 %	0.59 (0.50–0.70)	<0.001	30.5 %	1.00 (0.88–1.13)	1.0
		>15 years	516	18.0 %	7.8 %	0.43 (0.33–0.55)	<0.001	25.4 %	1.41 (1.19–1.66)	<0.001
	Year	2012	629	20.5 %	11.4 %	0.56 (0.47–0.66)	<0.001	19.7 %	0.96 (0.86–1.08)	0.58
		2013	535	19.3 %	9.7 %	0.50 (0.41–0.62)	<0.001	34.2 %	1.78 (1.51–2.09)	<0.001
Kihihi	Age categories	<5 years	345	18.0 %	8.4 %	0.47 (0.35–0.62)	<0.001	34.5 %	1.92 (1.60–2.31)	<0.001
		5–15 years	558	34.2 %	17.9 %	0.52 (0.45–0.60)	<0.001	41.6 %	1.21 (1.11–1.33)	<0.001
		>15 years	642	15.9 %	4.7 %	0.29 (0.21–0.41)	<0.001	21.5 %	1.35 (1.15–1.60)	<0.001
	Year	2012	787	22.7 %	11.7 %	0.51 (0.44–0.60)	<0.001	22.6 %	0.99 (0.90–1.09)	1.0
		2013	758	23.2 %	8.8 %	0.38 (0.31–0.46)	<0.001	41.0 %	1.77 (1.58–1.98)	<0.001
Nagongera	Age categories	<5 years	353	63.2 %	46.4 %	0.74 (0.68–0.80)	<0.001	75.1 %	1.19 (1.11–1.27)	<0.001
		5–15 years	752	80.2 %	58.1 %	0.72 (0.69–0.76)	<0.001	77.3 %	0.96 (0.93–1.00)	0.07
		>15 years	619	45.7 %	21.2 %	0.46 (0.41–0.53)	<0.001	35.2 %	0.77 (0.70–0.84)	<0.001
	Year	2012	804	60.3 %	43.7 %	0.72 (0.68–0.77)	<0.001	55.2 %	0.92 (0.87–0.97)	0.001
		2013	920	67.8 %	41.4 %	0.61 (0.57–0.65)	<0.001	67.4 %	0.99 (0.95–1.04)	0.82

^a^Parasite prevalence

^b^Prevalence ratio using PCR as the reference group

### Relationships between age and year with estimates of parasite prevalence using different diagnostic modalities

In all sites, parasite prevalence followed a similar and expected age-related pattern, peaking in children aged 5–15 years and declining in older participants (Table 4). The relative differences in parasite prevalence in children <5 years and those aged 5–15 years were greater for estimates determined by microscopy and PCR than for RDTs in all sites, as RDTs appeared to over-estimate parasite prevalence in younger children. Estimates of parasite prevalence by microscopy were consistent from 2012 to 2013, with no significant differences between the survey years in any site. However, parasite prevalence determined by RDT increased significantly in 2013 at all sites, especially in the two lower transmission sites, Walukuba and Kihihi. Estimates of parasite prevalence by PCR were consistent from 2012 to 2013 in Walukuba and Kihihi, but increased modestly from 2012 to 2013 in Nagongera (Table 4).

**Table 4:** Relationship between age and year with estimates of parasite prevalence using different diagnostic modalities

Study site	Covariate		N	Microscopy			RDT			PCR		
				PP^a^	PR^b^ (95 % CI)	p-value	PP^a^	PR^b^ (95 % CI)	p-value	PP^a^	PR^b^ (95 % CI)	p-value
Walukuba	Age categories	<5 years	284	6.3 %	Reference		22.9 %	Reference		9.9 %	Reference	
		5–15 years	364	18.1 %	2.86 (1.74–4.70)	<0.001	30.5 %	1.36 (1.05–1.76)	0.02	30.5 %	3.09 (2.10–4.54)	<0.001
		>15 years	516	7.8 %	1.23 (0.72–2.10)	0.45	25.4 %	1.12 (0.87–1.44)	0.39	18.0 %	1.83 (1.23–2.72)	0.003
	Year	2012	629	11.4 %	Reference		19.7 %	Reference		20.5 %	Reference	
		2013	535	9.7 %	0.90 (0.65–1.26)	0.54	34.2 %	1.75 (1.44–2.13)	<0.001	19.3 %	0.97 (0.77–1.22)	0.80
Kihihi	Age categories	<5 years	345	8.4 %	Reference		34.5 %	Reference		18.0 %	Reference	
		5–15 years	558	17.9 %	2.15 (1.45–3.18)	<0.001	41.6 %	1.16 (0.98–1.37)	0.09	34.2 %	1.91 (1.48–2.45)	<0.001
		>15 years	642	4.7 %	0.56 (0.34–0.92)	0.02	21.5 %	0.62 (0.51–0.76)	<0.001	15.9 %	0.88 (0.66–1.18)	0.40
	Year	2012	787	11.7 %	Reference		22.6 %	Reference		22.7 %	Reference	
		2013	758	8.8 %	0.76 (0.56–1.01)	0.06	41.0 %	1.76 (1.51–2.05)	<0.001	23.2 %	0.99 (0.83–1.19)	0.93
Nagongera	Age categories	<5 years	353	46.4 %	Reference		75.1 %	Reference		63.2 %	Reference	
		5–15 years	752	58.1 %	1.25 (1.10–1.42)	0.001	77.3 %	1.03 (0.97–1.11)	0.33	80.2 %	1.27 (1.17–1.39)	<0.001
		>15 years	619	21.2 %	0.46 (0.38–0.55)	<0.001	35.2 %	0.47 (0.42–0.54)	<0.001	45.7 %	0.73 (0.65–0.82)	<0.001
	Year	2012	804	43.7 %	Reference		55.2 %	Reference		60.3 %	Reference	
		2013	920	41.4 %	0.92 (0.84–1.02)	0.13	67.4 %	1.18 (1.11–1.26)	<0.001	67.8 %	1.10 (1.03–1.17)	0.003

^a^Parasite prevalence

^b^Prevalence ratio adjusted by other covariates

## Discussion

Cross-sectional surveys estimating parasite prevalence offer a practical method for malaria surveillance and are used to monitor changes over time and space [3, 10, 19, 20]. Parasite prevalence is used frequently as a proxy measure of transmission intensity; however, this measure has limitations as an indicator of malaria burden. Estimates of parasite prevalence may vary considerably depending on the diagnostic test used and the age-group being sampled. In addition, these variations may be further modified by the underlying transmission intensity and temporal factors independent of true changes in malaria burden. This study, compared estimates of parasite prevalence determined by microscopy and RDTs, to that determined by PCR (the gold standard) using samples collected from two consecutive annual community surveys in three areas of varying transmission intensity. Microscopy had limited but consistent sensitivity, which generally decreased with increasing age at all three study sites. Specificity of microscopy was very high, such that relative differences in estimates of parasite prevalence across different age groups, study sites, and study years followed expected patterns and were consistent with relative changes in estimates using PCR. The sensitivity of RDTs was higher than microscopy and also decreased with increasing age. However, in contrast to microscopy, specificity of RDTs varied considerably from 1 year to the next and had a complex relationship to age that varied across the sites. This resulted in estimates of parasite prevalence that did not follow the same age pattern as with microscopy and PCR and inaccurately reflected changes in prevalence, as assessed by PCR, from year to year.

Several diagnostic tests are available for detection of malaria parasitaemia, with microscopy being the mainstay of diagnosis. Microscopy is relatively inexpensive to perform, and can be used to differentiate malaria species and quantify parasitaemia, but has known limitations [21, 22]. In this study, microscopy was highly specific and parasite estimates were consistent irrespective of the age of the population studied, year and transmission intensity. These advantages make microscopy, when performed well, a reliable tool for monitoring disease burden in surveys over time. However, the low sensitivity compared to PCR, and resulting lower parasite prevalence estimates, need to be taken into account when interpreting results [23–25]. RDTs are increasingly being used independently or in combination to microscopy in surveys [8]. RDTs are attractive as diagnostic tools due to their higher sensitivity compared to microscopy, ease of use and rapid availability of results [14, 26]. However, specificity of RDTs and parasite prevalence estimates were highly variable in this study. This variability may affect the interpretation of prevalence estimates. According to the RDT results, parasite prevalence increased significantly from 2012 to 2013, suggesting an increase in the disease burden. However, these results were not consistent with estimates from microscopy and PCR, and other study findings conducted during the same time period [17]. Thus, relying on RDT results would have provided an inaccurate picture of the of the malaria burden in the study sites.

Malaria surveillance has typically targeted children aged 2–10 years for estimates of parasite prevalence [27]. The Roll Back Malaria Monitoring and Evaluation group recommends that national surveys target children under 5 years for parasitaemia and anaemia testing [8], while other groups have explored alternative target populations, such as school-aged children who may be more accessible [28, 29]. These results show that the parasite prevalence estimates vary markedly with age, increasing during childhood and then declining following adolescence. This observed pattern has been well-described in malaria endemic countries [30], but the shape of the age-parasite prevalence curve is modified by the underlying transmission intensity [16]. Thus, when selecting a given age group for estimating disease burden, it should be acknowledged that survey results may over- or under-estimate parasite prevalence, when compared to the wider population [29]. This also highlights the importance of consistently estimating parasite prevalence in the same age group when monitoring malaria burden and the impact of control interventions over time.

In this study, the performance of the diagnostic tests varied with changing transmission intensity and age. The sensitivity of microscopy was highest in the highest transmission setting, consistent with the observation that the relative proportion of sub-microscopic infections, those below the level of detection by microscopy, is higher in lower transmission settings [12, 31]. The parasite densities of asymptomatic infections will vary with the level of acquired immunity, which is dependent on the transmission setting and age [32, 33]. For example, in higher transmission settings, recurrent malaria infections lead to earlier, and greater, age-specific acquired immunity such that individuals are more likely to tolerate high-density malaria infections without developing symptoms [34–36]. The lower sensitivity of microscopy in younger children could also be due to a higher proportion of these children having been treated recently for malaria, resulting in very low-density parasites remaining from a prior treatment.

Population surveys commonly use traditional diagnostic techniques including microscopy or RDTs which may miss low-grade infections that are below the level of detection of these tools (sub-patent infections). Studies in high transmission areas have shown that as many as two-thirds of microscopy-negative patients may have sub-patent malaria infections [37–40]. Molecular techniques, such as PCR, are more sensitive, and thus are more likely to detect sub-patent infections [41]. However, PCR must be performed by highly trained technicians in sophisticated laboratories, which makes this method more expensive and less feasible for large-scale surveys. Recently, loop-mediated isothermal amplification (LAMP) has been optimized for the rapid amplification and detection of parasite DNA [42, 43]. LAMP testing is highly sensitive and can be performed in minimally equipped laboratories by technicians after a brief training period [41, 44], which makes it an attractive alternative to PCR for endemic areas, and a potential option for population-based surveys.

This study was not without limitations. First, a significant variation in the specificity of RDTs was observed between 2012 and 2013 despite using the same brand of RDTs and the same survey staff in both surveys, and in the absence of any major control interventions within the 2 years. The cause of the variation between the 2 years could not be established; however, it is speculated that the performance of the RDTs could have been affected by transportation or storage conditions, or possibly changes in seasonality. Second, PCR was not performed on samples that were positive by both microscopy and RDT; however, it was assumed that PCR would be positive if both microscopy and RDT results were positive and that this cost-saving measure did not affect the study findings.

## Conclusion

Parasite prevalence estimates varied according to the diagnostic test employed, the age of the individual tested and the transmission intensity of the area. When planning for population-based community surveys, it is important to recognize the importance of the target age group, the survey site, and the choice of the diagnostic test, and their potential impact on estimates of parasite prevalence. Finally, these results suggest that RDTs may not be the optimal test for cross-sectional surveys of asymptomatic populations because of the high variability in parasite prevalence estimates based on this method.
